# Exogenous *Streptococcus pneumoniae* Endophthalmitis in Diabetic Rabbits

**DOI:** 10.1038/srep46196

**Published:** 2017-04-07

**Authors:** Angela H. Benton, Linda K. Fulton, Mary E. Marquart

**Affiliations:** 1 University of Mississippi Medical Center, Department of Microbiology and Immunology, Jackson, Mississippi 39216, USA; 2 University of Mississippi Medical Center, Center for Comparative Research, Jackson, Mississippi 39216, USA

## Abstract

Diabetics are at increased risk for eye infections including bacterial endophthalmitis. It is unclear whether the severity of endophthalmitis is greater in these patients due to confounding factors such as pre-existing ocular diseases in some but not others. Therefore, we tested the hypothesis that disease severity and/or bacterial loads would be significantly higher in a Type I diabetic rabbit model of *Streptococcus pneumoniae* endophthalmitis. Rabbits were treated with alloxan to destroy pancreatic islet cells, or mock-treated with vehicle, and maintained for 10 days before intravitreal infection with *S. pneumoniae* E353. Clinical scoring of the eyes was performed 24 and 48 hours after infection, followed by euthanasia and vitreous harvest to quantitate bacterial loads. There were no significant differences in clinical scores (P ≥ 0.440) or bacterial loads (P = 0.736), however, 4/12 (33%) of the diabetic rabbits became bacteremic. This finding not only indicates a breakdown in the blood-ocular barrier, but also prompts further investigation into the exploitation of the diabetic eye by the streptococci.

Individuals with systemic conditions such as diabetes are more susceptible to infections than healthy individuals. One such example is bacterial endophthalmitis, an inflammatory infection of the interior of the eye. The two major interior cavities of the eye are the aqueous humor and the vitreous humor. Diabetics are susceptible to both the endogenous (metastatic) and exogenous forms of this disease^1^,^2^,^3^,^4^,^5^. Endogenous bacterial endophthalmitis occurs when bacteria from another body site travel to the eye to cause an intraocular infection. Exogenous endophthalmitis cases, caused by bacteria entering the eye from the exterior of the body, can occur following trauma or procedures such as cataract surgeries or surgeries necessitating corneal sutures^1^,^3^,^4^,^6^. The most common bacteria isolated as causes of post-operative endophthalmitis are coagulase-negative staphylococci, *Pseudomonas*, and streptococcal species^6^,^7^,^8^. Culture-proven cases not necessarily linked to surgery include *Staphylococcus aureus* in addition to coagulase-negative staphylococci and viridans group streptococci^9^.

Streptococcal species of the oral cavity are three times more likely to be isolated in post-injection endophthalmitis, where bacteria are introduced into the eye from intraocular injections of therapeutic drugs for other ocular diseases^10^. Streptococci are associated with poor visual outcomes in endophthalmitis such as lack of light perception^6^,^11^ and loss of the globe^12^,^13^ despite antibiotic treatment and/or pars plana vitrectomy (removal of the infected vitreous humor). In post-injection endophthalmitis preventive application of topical antibiotics does not result in decreased infection rates, as indicated by retrospective studies. It is suspected the use of intravitreal and topical antibiotics could permit bacterial mutagenesis, leading to a higher rate of infection and more severe outcome^14^.

The Centers for Disease Control and Prevention estimated that 9.3% of Americans have diabetes as of 2014^15^. Diabetes is not only associated with systemic health problems but also linked to a variety of ocular diseases that require intraocular surgery or intravitreal injections^16^. In a study of endophthalmitis associated with ocular blebs that are created during surgery to promote proper drainage of ocular fluids, streptococci were isolated as the causes of 50% of the culture-positive cases, and a predictor for better visual outcome was the absence of diabetes^17^. The French Institutional Endophthalmitis Study Group reported an association between diabetic patients that had undergone cataract surgery and *Streptococcus* species as the isolated agent causing endophthalmitis^18^. The authors of a 2012 literature review concluded that diabetics were more predisposed to bacterial endophthalmitis following ocular surgery, based on previous studies reporting 14–21% of postoperative patients as having diabetes^19^.

Large epidemiological studies of bacterial endophthalmitis have reported general trends toward worse visual outcomes for diabetics versus non-diabetics^4^,^20^. A confounding factor in analyzing the data from those studies is the existence of diabetic eye disease (e.g., retinopathy) in some patients but not others. To determine whether diabetes is associated with more severe disease, we chose a rabbit model of alloxan-induced diabetes to examine exogenous *S. pneumoniae* endophthalmitis. We hypothesized that clinical scores and/or bacterial loads would be significantly higher for the eyes of diabetic rabbits compared to those of non-diabetic rabbits.

## Results

Two independent experiments were performed with a starting number of 12 rabbits in each experiment (7 diabetic, 5 control per experiment). One of the diabetic rabbits in the first experiment lost more than 20% body weight and was euthanized as a humane endpoint. Two additional diabetic rabbits in the first experiment, and one in the second experiment, became moribund shortly before the 48-hour clinical examination and were euthanized; blood and vitreous were collected from these rabbits to quantify bacterial loads, but 48-hour clinical examinations were not possible. One of the diabetic rabbits in the second experiment had fluctuating blood glucose measurements throughout the study and had a measurement of 231 mg/dL (less than 300) by the time of the infection, and was therefore excluded from the study. Both experiments yielded statistically similar results, and the data from the 2 experiments were combined for analysis (n = 12 diabetic, n = 10 control for 24-hour clinical examination and 48-hour bacterial loads, and n = 9 diabetic, n = 10 control for 48-hour clinical examination).

**Figure 1:**
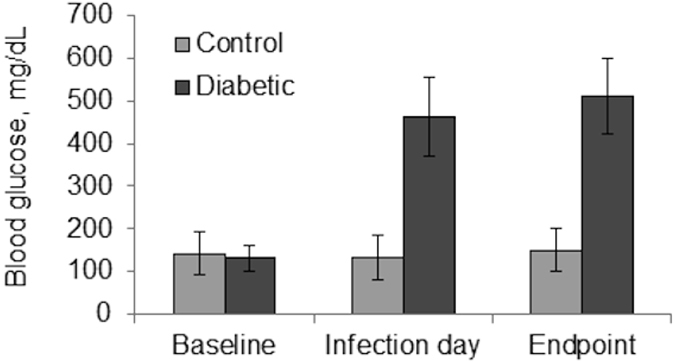
Blood glucoses of both diabetic and control rabbits were measured using a glucose meter. Values were averaged and analyzed; baseline, p = 0.501; infection day and endpoint, p < 0.001. Error bars represent standard deviation.

All rabbits had similar blood glucose concentrations prior to the start of the study (Fig. 1, baseline, p = 0.501; Cohen’s d = 0.298). By the day of the infection, and then 48 hours later at euthanasia, rabbits in the diabetic group had significantly higher blood glucose concentrations than control rabbits (Fig. 1, infection day and endpoint, p < 0.001; Cohen’s d = 4.334 and 5.027 for infection day and endpoint, respectively).

**Figure 2:**
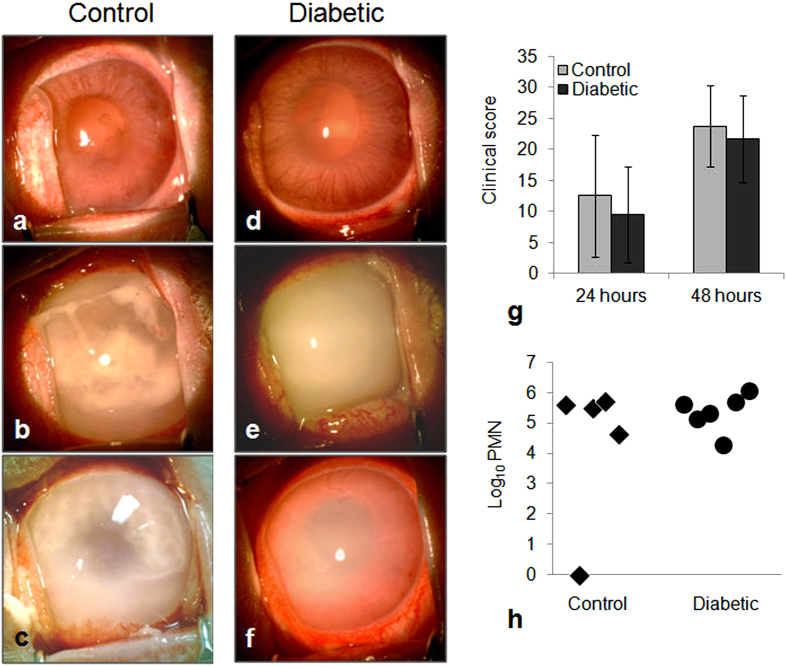
(**a**–**f**) Eyes of control (**a**–**c**) and diabetic (**d**–**f**) rabbits, 48 hours after infection. (**g**) Clinical scores (determined by slit lamp examination) of control and diabetic rabbits; p ≥ 0.440. (**h**) Quantification of PMNs (determined by myeloperoxidase activity) in vitreous humor of both control and diabetic rabbits 48 hours after infection; p = 0.394. Error bars in (**g**) represent standard deviation.

The actual inocula for the two separate experiments were 833 CFU and 616 CFU as determined by serial dilution and plate counts. Infected eyes from both groups showed variability in the severity of disease, as shown for 48 hours post-infection in Fig. 2a–f (24 hours not shown). Some eyes had intense inflammation extending into the aqueous cavity whereas others had mild vitreous inflammation and only partial loss of red reflex (the red color observed through the pupil when a light is shined directly into the eye). Mean clinical scores were similar for both groups at both time points (Fig. 2g; p ≥ 0.440; Cohen’s d = 0.343 and 0.300 at 24 and 48 hours, respectively). The quantity of polymorphonuclear neutrophils (PMNs) present in a subset of rabbit vitreous samples at 48 hours, as determined by myeloperoxidase activity, was also similar for both groups (Fig. 2h; p = 0.394; Cohen’s d = 0.594).

**Figure 3:**
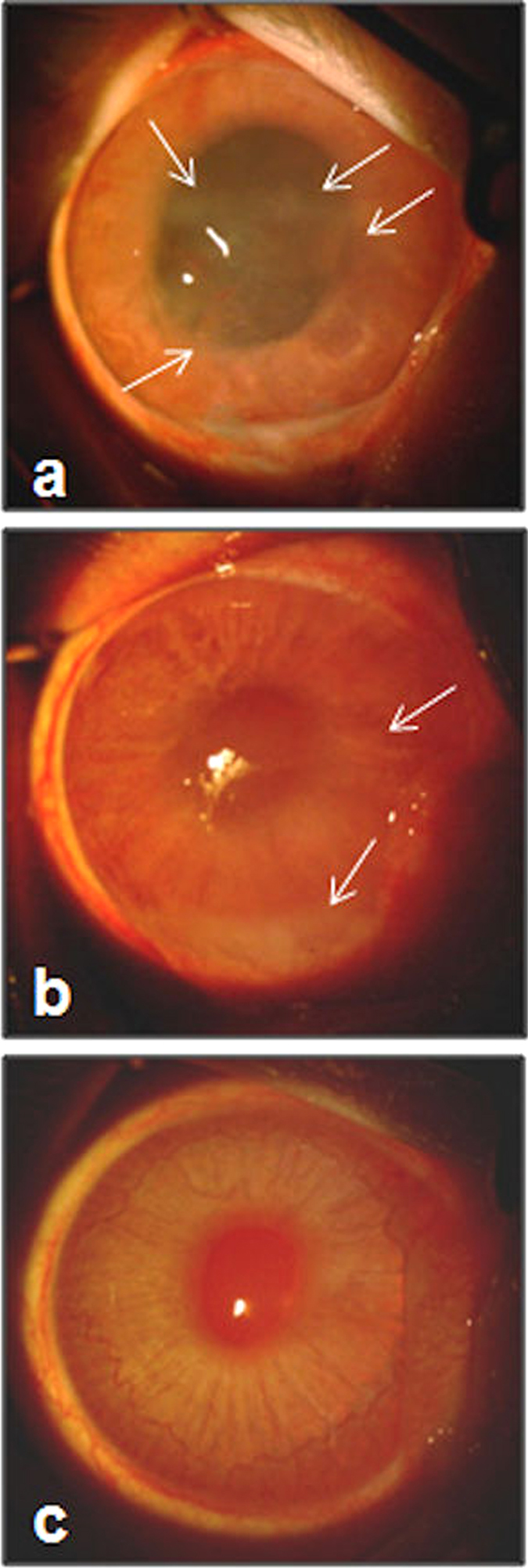
Inflammation (indicated by white arrows) of uninfected, contralateral eyes of diabetic rabbits (**a**,**b**). (**c**) Representative photograph of an uninfected contralateral eye of a control rabbit.

**Figure 4:**
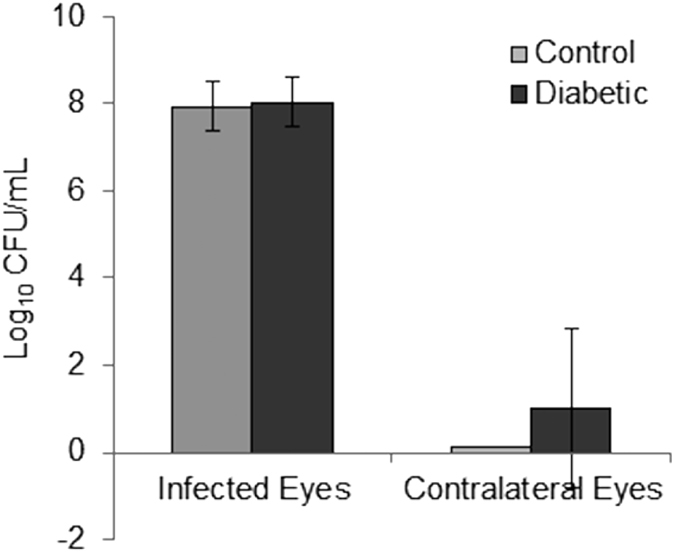
Log CFU/mL of bacteria recovered from infected and contralateral eyes after 48 hours after infection for control and diabetic rabbits (infected, p = 0.736; contralateral, p = 0.084). Error bars represent standard deviation.

Inflammation was observed in some of the uninfected contralateral eyes of the diabetic rabbits, but not the control rabbits, 48 hours after infection (Fig. 3). Clinical scoring was not performed for these eyes, however, vitreous from the uninfected eyes was collected, serially diluted, and plated after euthanasia to determine whether cross-infection occurred from the infected eyes. These quantitations included 3 contralateral eyes from diabetic rabbits that were moribund and were euthanized just prior to the 48-hour clinical examination. Of the 12 diabetic rabbits, 3 (25%) had culture-positive vitreous in the contralateral eye resembling S. *pneumoniae* on blood agar (alpha-hemolytic colonies) with no other apparent colony types. Two of these 3 rabbits were the moribund ones that were euthanized without a final clinical examination. The bacterial loads for the contralateral eyes of these 3 rabbits were 8.167 × 10^3^, 4.333 × 10^4^, and 3.833 × 10^3^ CFU/mL. All of the contralateral eyes in the control group of rabbits were culture-negative. There was no significant difference between diabetic and non-diabetic rabbits in vitreous bacterial loads of the infected eyes (Fig. 4, p = 0.736; Cohen’s d = 0.151), nor was there a significant difference for the contralateral eyes (p = 0.084), although the effect size for the contralateral eyes was large (Cohen’s d = 0.776).

The diabetic rabbits appeared in good health (with the exception of the one that lost more than 20% of its starting weight) until after the vitreous infection with *S. pneumoniae*. As previously stated, 3 diabetic rabbits became moribund shortly before the 48-hour clinical examination and were euthanized as a humane endpoint. To determine whether a breach in the retinal-blood barrier occurred, the blood of all rabbits was serially diluted and plated to determine the possibility of the presence of bacteria in the bloodstream. Four of 12 diabetic rabbits (33%) had culture-positive blood resembling *S. pneumoniae* on blood agar with no other apparent colony types. Two of the blood samples produced colonies that were too numerous to count at the highest dilution plated, translating to greater than 10^8^ CFU/mL in the blood. The other 2 blood samples had 1.267 × 10^5^ and 1.50 × 10^2^ CFU/mL. These culture-positive rabbits included the 3 moribund rabbits. All of the control rabbits had culture-negative blood.

## Discussion

The major findings of this study were that clinical endophthalmitis scores and vitreous bacterial loads were not significantly different between diabetic and non-diabetic rabbits. However, one-third of the diabetic animals became bacteremic. This latter result is likely due to a weakened blood-ocular barrier which is common for diabetes^21^. Moreover, one-fourth of the diabetic animals developed infections in their contralateral eyes, likely due to the bacteremia. Diabetics have increased vascular permeability and angiogenesis due to vascular endothelial growth factor (VEGF), and some diabetic patients undergo intravitreal injections of bevacizumab, an inhibitory antibody to VEGF, to treat macular edema (an inflammation of the central part of the retina called the macula)^22^,^23^,^24^. A role for this increased vascular permeability has been determined for endogenous bacterial endophthalmitis (EBE), which is an infection of the vitreous from bacteria that infected a different body site and transported to the eye through the bloodstream. Also, structural changes within the diabetic eye are indicated by an association of increased vascular permeability of the blood-ocular barrier and increased occurrence of EBE^21^.

The study described herein used an acute diabetes animal model in which the rabbits were infected a relatively short time after inducing Type I diabetes. Our choice of this acute model was based partly on the knowledge that *S. pneumoniae* utilizes glucose as a primary carbon source for growth^25^. It is well-documented that diabetics have elevated glucose in the vitreous humor mimicking blood glucose measurements^26^,^27^. *S. pneumoniae* is also able to utilize mannose, galactose, and N-acetylglucosamine. Each of these sugars can be processed to yield substrates that are necessary for the glycolytic pathway^25^,^28^. However, as opposed to glucose, these carbohydrates result in attenuated growth^28^. This finding is underscored by the preferential uptake of glucose when introduced to cultures containing any alternate carbohydrates^25^.

We verified that the diabetic rabbits had elevated glucose concentrations in the vitreous (≥600 mg/dL compared to approximately 185 mg/dL in control rabbits) at the time of infection, and reasoned that the bacteria would be more metabolically fit in that high-glucose environment. Our results, however, suggest that increased glucose in the vitreous does not have a significant effect on disease severity or bacterial growth. Overall, the vitreous environment regardless of glucose concentration appeared to provide an optimal medium for bacterial fitness, however, the specific components of the vitreous enabling bacterial growth are unknown.

To examine whether chronic diabetes results in more severe endophthalmitis, long-term maintenance of diabetic animals would be warranted. Likewise, examination of endophthalmitis in Type 2 diabetes with or without metabolic disease would require a separate model system than that described here. Age, sex, and animal strain should also be considered to investigate correlations between diabetes and development of bacteremic infections from exogenous endophthalmitis.

## Methods

### Bacterial strain and culture conditions

*Streptococcus pneumoniae* E353, a capsule type 6 A human endophthalmitis strain, was kindly provided by Regis P. Kowalski (Charles T. Campbell Eye Microbiology Laboratory, University of Pittsburgh, Pittsburgh, Pennsylvania, USA). Frozen stocks of E353 were routinely cultured for isolation on sheep’s blood agar (Oxoid, Basingstoke, Hampshire, England) for 18–24 hours at 37 °C and 5% CO_2_. Isolated colonies were inoculated into Todd Hewitt (Bacto^TM^) broth containing 0.5% yeast extract (THY) and then incubated for approximately 18 hours at 37 °C and 5% CO_2_. These 18-hour cultures were then diluted 100-fold in fresh THY and incubated until the optical density at 600 nm reached 0.23, which corresponded to approximately 1 × 10^7^ colony-forming units per mL (CFU/mL) as determined by previous growth curve analysis. Serial 10-fold dilutions of this logarithmic-phase subculture were prepared in sterile PBS (1.64 M NaCl: 2.3 × 10^−3^ M NaH_2_PO_4_: 7.7 × 10^−3^ M Na_2_HPO_4_, pH 7.0) and the third dilution (corresponding to a target of 10^2^ CFU per 10 μL) was used for inoculation. Serial dilutions were also plated on blood agar to verify purity and quantity.

### Animals

Twenty-four 2 kg specific-pathogen-free female New Zealand white rabbits (strain code 571) were purchased from Charles River (Oakwood Research facility, Michigan, USA). Rabbits were socially housed and given standard chow and drinking water *ad libitum*. Enrichment included plastic chew toys, jingle bells, plastic balls, daily petting, and hay as a treat. Animal procedures were reviewed and approved by the University of Mississippi Medical Center’s Institutional Animal Care and Use Committee. All animal experiments were performed in accordance with the tenets of the Association for Research in Vision and Ophthalmology.

### Study Design

Two independent experiments with 12 rabbits per experiment were done. For each experiment, 5 rabbits were assigned to the control group and 7 rabbits were assigned to the diabetic group. More rabbits were chosen for the diabetic group to account for possible attrition in this group.

Induction of Type I diabetes was done similarly to the method of Wang *et al*.^29^, except that the current study was not long-term. Several days after arrival and acclimation, baseline weights and blood glucose measurements were taken. Blood glucose measurements were done using a standard diabetic glucose meter and strips, and one drop of blood was obtained by pricking the lateral ear vein with a 30-gauge needle. Each rabbit was then given 0.5–1.0 mg/kg acepromazine subcutaneously, and ear fur was shaved. Topical 5% lidocaine/prilocaine cream was applied to the lateral ear vein of the shaved ear 15 to 30 minutes prior to injection. Each control rabbit received an intravenous injection of 2 mL per kg sterile filtered PBS slowly over the course of 2 minutes. Each diabetic group rabbit received an intravenous injection of 2 mL per kg sterile filtered 5% alloxan monohydrate (Sigma-Aldrich, St. Louis, Missouri, USA) in PBS (prepared fresh) over 2 minutes.

Alloxan-treated rabbits have been reported to develop severe hypoglycemia within the first few days after injection^29^. To prevent hypoglycemia in the alloxan-treated rabbits, 10 mL of sterile filtered 5% pharmaceutical-grade glucose in PBS was injected subcutaneously into all of these rabbits 4 hours after alloxan administration. Eight hours after alloxan administration, blood glucose measurements were taken, and subcutaneous glucose was administered to any rabbit whose glucose was less than 100 mg/dL. Those rabbits with low blood glucose were then given another dose of subcutaneous glucose 12 hours post-alloxan injection.

Blood glucose measurements were taken twice daily for the following 3 days, and then once daily for another 7 days. During this time, diabetes was managed by administration of subcutaneous pharmaceutical-grade insulin (Humulin, rDNA, Indianapolis, Indiana, USA) diluted in sterile filtered PBS once daily in the following quantities: 1 U/kg for blood glucose of 350–400 mg/dL, 2 U/kg for 400–500 mg/dL, or 3 U/kg for 500–600 mg/dL. Body weights were also recorded every other day during this time. Based on the study of Wang *et al*.^29^, who administered a second alloxan dose to any rabbit not maintaining at least 300 mg/dL blood glucose, we chose 300 mg/dL as the minimum required concentration for a rabbit to proceed to the infection. We opted not to administer a second dose of alloxan due to its high toxicity.

Each rabbit was anesthetized with a subcutaneous injection of 35–50 mg/kg ketamine and 2–10 mg/kg xylazine, and topical 0.5% proparacaine was applied to one eye. *S. pneumoniae* E353 was injected into the vitreous humor of the anesthetized eye of each rabbit at a target concentration of 10^2^ CFU in a volume of 10 μL. Clinical examination was done 24 and 48 hours after infection by an observer who was masked to the grouping of each rabbit. Clinical severity scores were assigned according to previously published methods^30^,^31^. In short, 8 parameters were graded from 0 to 4 and grades were totaled, yielding an overall score with a theoretical maximum of 32. Each rabbit was then euthanized while under anesthesia with an intravenous overdose of sodium pentobarbital, and blood as well as vitreous from both eyes was harvested. Vitreous and blood were serially diluted and plated on blood agar to quantify bacterial loads. A subset of vitreous was tested for myeloperoxidase activity to assess neutrophil (PMN) activity according to the method of Williams *et al*.^32^.

### Statistics

Sample sizes were determined a priori to provide a minimum of 90% power based on the pre-experimental assumptions that (1) mean clinical scores would differ by 8 with standard deviations of 5, and (2) mean bacterial loads would differ by 2 log_10_ CFU/mL with standard deviations of 0.5, at an alpha level of 0.05 (5% probability of Type I error). All quantitative data are expressed as the mean ± standard deviation. Following a one-way analysis of variance to detect overall differences, pairwise comparisons were done using a two-tailed t-test. All comparisons were between control animals and diabetic animals at a given time point. P-values and effect sizes (Cohen’s d) were recorded.

## Additional Information

**How to cite this article**: Benton, A. H. *et al*. Exogenous *Streptococcus pneumoniae* Endophthalmitis in Diabetic Rabbits. *Sci. Rep.* **7**, 46196; doi: 10.1038/srep46196 (2017).

**Publisher's note:** Springer Nature remains neutral with regard to jurisdictional claims in published maps and institutional affiliations.
